# Necrotizing (Abscessing) Lymphadenopathy and the Diagnostic Value of Contrast-Enhanced Ultrasound (CEUS): A Review with Clinical Vignettes

**DOI:** 10.3390/diagnostics16060888

**Published:** 2026-03-17

**Authors:** Christian Görg, Yi Dong, Görg Friedemann, Christian Jenssen, Michael Kallenbach, Kathleen Möller, Findeisen Hajo, Nitin Chaubal, Christoph Frank Dietrich

**Affiliations:** 1 Interdisciplinary Center of Ultrasound Diagnostics, Gastroenterology, Endocrinology, Metabolism and Clinical Infectiology, University Hospital Giessen and Marburg, Philipp University of Marburg, Baldingerstraße, 35043 Marburg, Germany; goergc53@gmail.com (C.G.); f.goerg@gmx.de (G.F.); 2 Department of Ultrasound, Xinhua Hospital Affiliated to Shanghai Jiaotong University School of Medicine, Shanghai 200082, China; drdaisydong@hotmail.com; 3 Department for Internal Medicine, Krankenhaus Märkisch Oderland, 15344 Strausberg, Germany; c.jenssen@khmol.de; 4 Brandenburg Institute for Clinical Ultrasound (BICUS), Brandenburg Medical University, 16816 Neuruppin, Germany; 5 Klinik für Gastroenterologie, Hepatologie und Infektiologie, Universitätsklinikum Düsseldorf, 40225 Dusseldorf, Germany; michael.kallenbach@med.uni-duesseldorf.de; 6 Medical Department I/Gastroenterology, SANA Hospital Lichtenberg, 10365 Berlin, Germany; k.moeller@live.de; 7 Department for Hematology, Oncology and Immunology, University Hospital Giessen and Marburg, Philipp University of Marburg, 35037 Marburg, Germany; 8 Thane Ultrasound Centre, Jaslok Hospital and Research Centre, Mumbai 400026, India; 9 University Hospital Frankfurt, Johann-Wolfgang-Goethe University Frankfurt on the Main, Theodor-Stern-Kai 7, 60596 Frankfurt am Main, Germany

**Keywords:** contrast-enhanced ultrasound, CEUS, lymphadenopathy, necrosis, abscess, infection, malignancy

## Abstract

Necrotizing (abscessing) lymphadenopathy is a clinically relevant condition with a broad differential diagnosis, including acute bacterial infections, mycobacterial disease, zoonoses, fungal and parasitic infections, autoimmune disorders, and malignancies with central necrosis. Early and reliable differentiation between these causes is important to avoid misdiagnosis and to guide appropriate therapy. This review summarizes the pathophysiological mechanisms, typical imaging features, and diagnostic value of contrast-enhanced ultrasound (CEUS) in necrotizing lymphadenopathy. Representative clinical vignettes illustrate the disease spectrum and correlate CEUS patterns with underlying pathology. The literature review was narrative and based on targeted searches of PubMed/MEDLINE and Google Scholar focusing on CEUS in necrotizing lymphadenopathy. A brief literature overview highlights current evidence, limitations, and research gaps. Conventional B-mode ultrasound (BMUS) and Doppler typically demonstrate enlarged hypoechoic or heterogeneous nodes with reduced central vascularity but lack specificity for necrosis. CEUS provides real-time visualization of nodal microvascular perfusion, which may support clearer differentiation between viable tissue and necrotic or abscess cavities. Common but non-specific CEUS patterns include central non-enhancement with a peripheral hyperemic rim in abscesses, irregular avascular cores in tuberculous lymphadenopathy, patchy non-enhancing areas in autoimmune conditions, and heterogeneous enhancement with ill-defined necrosis in malignant nodes. CEUS can support biopsy targeting, facilitate drainage procedures, and enable radiation-free follow-up. CEUS may offer diagnostic and interventional advantages in the evaluation of necrotizing lymphadenopathy, offering more consistent characterization of nodal necrosis compared with conventional sonography. While most evidence focuses on tuberculosis and malignancy, growing experience with zoonotic and autoimmune diseases suggests broader utility. Most currently available evidence derives from observational studies and small case series, highlighting the need for prospective multicenter validation. Standardization of CEUS criteria, integration into multiparametric ultrasound protocols, and multicenter validation are needed to establish CEUS as a routine component in the diagnostic work-up of necrotizing lymphadenopathy.

## 1. Introduction

Lymphadenopathy is a common clinical finding and represents a diagnostic challenge across many medical disciplines. Among its various forms, necrotizing or abscessing lymphadenopathy is of particular relevance, as it may result from infectious or malignant causes that often mimic one another [1,2,3,4,5]. Accurate differentiation between malignant necrotic nodes and abscesses is essential to prevent misdiagnosis, unnecessary invasive procedures, or delays in initiating appropriate therapy.

Conventional imaging modalities, particularly B-mode ultrasound (BMUS) and Doppler sonography, are routinely used for the initial evaluation of enlarged lymph nodes [6,7,8]. BMUS can demonstrate enlarged, hypoechoic lymph nodes with heterogeneous echotexture and central liquefaction, whereas Doppler imaging often reveals reduced or absent central vascularity. However, these findings are nonspecific and may not reliably distinguish necrotic tissue from viable parenchyma [9,10].

Contrast-enhanced ultrasound (CEUS) has emerged as a valuable adjunct in this diagnostic setting [9,10]. CEUS may allow a more precise characterization of lymph nodes by depicting microvascular perfusion in real time. Non-enhancing central areas can be identified as necrotic or abscess cavities, whereas peripheral rim enhancement typically reflects inflammatory hyperemia. Moreover, CEUS can detect residual viable nodal tissue and reveal heterogeneous enhancement patterns suggestive of malignancy.

In addition to potentially improving diagnostic confidence, CEUS provides guidance for biopsy or aspiration, facilitates treatment decisions, and can be used to monitor therapeutic response [11,12].

Given the diverse underlying causes—including bacterial and mycobacterial infections, cat-scratch disease, tularemia, autoimmune disorders such as Kikuchi–Fujimoto disease, and malignant processes—the recognition and accurate characterization of necrotizing lymphadenopathy are essential.

This review aims to provide an integrated overview of the clinical and pathological mechanisms underlying necrotizing lymphadenopathy, summarize characteristic CEUS features, and illustrate their diagnostic relevance through representative clinical vignettes.

### 1.1. Methodology and Literature Search

The literature search for this review was performed in a narrative, non-systematic manner. Relevant publications were identified through targeted searches of PubMed/MEDLINE and Google Scholar, focusing on contrast-enhanced ultrasound (CEUS), necrotizing or abscessing lymphadenopathy, and related infectious, autoimmune, and malignant etiologies. Search terms included combinations of “contrast-enhanced ultrasound”, “CEUS”, “lymphadenopathy”, “necrosis”, “abscess”, “tuberculous lymphadenitis”, and “malignant lymph nodes”. Reference lists of key articles and current guidelines were additionally screened to identify further relevant studies. Given the heterogeneous nature of the available literature—largely consisting of observational studies, small series, and case reports—no formal systematic review or meta-analysis was undertaken.

### 1.2. CEUS Technique for Lymph Node Evaluation

CEUS was performed using a low-mechanical index (MI ≤ 0.10–0.12) technique in accordance with EFSUMB recommendations. A second-generation sulfur hexafluoride-based contrast agent (e.g., SonoVue^®^/Lumason^®^) was administered intravenously as a bolus (1.0–2.4 mL), followed by a 5–10 mL saline flush. Continuous real-time imaging was initiated immediately after injection and maintained for 60–120 s to assess arterial and early parenchymal phases; in selected cases, observation was extended up to 3–5 min to confirm persistent non-enhancement. Cine loops of 10–20 s were stored during key phases. Regions of interest were placed over enhancing and non-enhancing areas when required for qualitative comparison. For optimal reproducibility, the focal zone was positioned at or just below the lymph node, gain was adjusted to suppress background tissue signal, and excessive transducer pressure was avoided. High-MI imaging modes were not used immediately before or during CEUS acquisition to prevent microbubble destruction. Interpretation focused on enhancement distribution and the identification of avascular areas corresponding to necrosis or abscess formation, in correlation with B-mode, Doppler findings, and clinical context.

In this review, the term “necrotizing lymphadenopathy” is used as the overarching descriptor. The term “abscessing” is reserved for cases with liquefactive infectious necrosis.

Some figures illustrate representative or comparative imaging examples intended to demonstrate typical CEUS patterns; in these cases, complete clinical metadata are not available or not applicable.

### 1.3. Pathophysiology of Necrotizing (Abscessing) Lymphadenopathy

Lymphadenopathy reflects an adaptive response of the lymphatic system to various pathophysiological stimuli, including infectious agents, autoimmune reactions, and malignant infiltration [13]. When necrosis or abscess formation occurs within lymph nodes, it usually indicates advanced infection, specific inflammatory processes, or rapidly proliferating tumors that exceed their vascular supply. Understanding these mechanisms is essential for accurate interpretation of imaging findings and appropriate clinical management.

### 1.4. Infectious Causes

Pyogenic bacterial infections (e.g., *Staphylococcus aureus*, *Streptococcus pyogenes*, anaerobes) can lead to acute lymphadenopathy. Rapid bacterial proliferation and neutrophilic infiltration result in liquefactive necrosis and abscess cavity formation within the node. On imaging, this corresponds to centrally non-enhancing areas surrounded by peripheral rim hyperemia on CEUS.

Mycobacterial infections (tuberculosis and atypical mycobacteria) induce granulomatous inflammation with caseous necrosis. Over time, central acellularity and progressive liquefaction develop, resulting in the characteristic avascular necrotic cores seen on CEUS, often accompanied by irregular rim enhancement representing active granulomatous tissue [14,15,16,17,18].

Zoonotic and atypical bacterial infections—including *Bartonella henselae* (cat-scratch disease) [19,20,21,22], *Francisella tularensis* (tularemia) [23,24,25,26], and *Yersinia enterocolitica* or *Yersinia pseudotuberculosis* [27,28,29,30,31,32]—typically reach lymph nodes via lymphatic spread from cutaneous or mucosal entry sites. The resulting granulomas show CEUS features comparable to pyogenic infections, but they are often multifocal and associated with systemic manifestations.

Brucellosis may occasionally cause necrotizing lymphadenopathy, particularly in endemic regions. The disease mechanism involves intracellular replication of *Brucella* species with granulomatous inflammation and focal necrosis, although large abscesses are uncommon [33,34,35,36].

### 1.5. Opportunistic Infections and Fungi

In immunocompromised patients, opportunistic pathogens such as *Histoplasma capsulatum* and *Cryptococcus neoformans* may cause necrotizing lymphadenopathy. These infections arise due to impaired host defense and uncontrolled intracellular fungal proliferation. On CEUS, avascular areas with irregular margins are typically observed, often in conjunction with systemic organ involvement.

### 1.6. Parasitic and Viral Associations

Although rare, toxoplasmosis can occasionally produce focal necrosis within lymph nodes due to destruction of lymphoid follicles by tachyzoites. Leishmaniasis may also involve deep abdominal or cervical lymph nodes, sometimes with necrotic degeneration in advanced stages. Viral infections such as Epstein–Barr virus or HIV typically cause reactive hyperplasia but may predispose to secondary necrosis when complicated by secondary bacterial superinfection [8,37].

### 1.7. Autoimmune and Inflammatory Disorders

Kikuchi–Fujimoto disease (histiocytic necrotizing lymphadenopathy) and systemic lupus erythematosus (SLE)-associated lymphadenopathy are representative non-infectious causes of necrotizing lymphadenopathy [3,5,38,39,40,41,42]. In Kikuchi–Fujimoto disease, apoptotic cell death mediated by cytotoxic T cells leads to patchy necrosis without neutrophilic infiltration, which explains the absence of liquefied abscess cavities and the CEUS finding of irregular, patchy non-enhancing areas rather than rim-enhancing abscesses. In SLE, immune-complex deposition and secondary small-vessel vasculitis may result in focal necrotic changes within affected lymph nodes.

### 1.8. Malignant Etiologies

Rapidly growing tumors—such as high-grade lymphomas or metastatic squamous cell carcinoma—may exceed their vascular supply, resulting in central ischemic necrosis. In contrast to infectious abscesses, these necrotic regions are surrounded by viable tumor tissue rather than an inflammatory capsule. On CEUS, this typically appears as irregular, heterogeneous enhancement with ill-defined non-enhancing areas, which may aid in distinguishing malignant from benign lymphadenopathy [9,10].

## 2. Clinical Correlation

**Figure 1:**
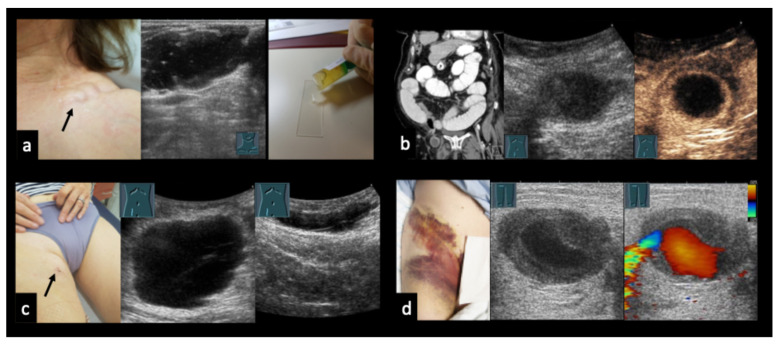
Examples of cystic-appearing, non-lymphatic masses. (**a**) Cystic-appearing ganglion arising from the left sternoclavicular joint; aspiration yielded clear, gelatinous fluid. (**b**) Right-sided incarcerated inguinal hernia on CT with a cystic-appearing inguinal structure on B-mode ultrasound. CEUS at 30 s shows absent central enhancement. (**c**) Right-sided postoperative mass presenting as anechoic on ultrasound; decompressive aspiration revealed serous fluid consistent with a postoperative seroma. (**d**) Right-sided groin mass after cardiac catheterization; B-mode ultrasound shows a hypoechoic lesion with a central anechoic area, and color Doppler confirms the diagnosis of a pseudoaneurysm. Arrow: location of the ultrasound finding.

When a space-occupying lesion appears sonographically compatible with necrotizing or abscessing lymphadenopathy, it is essential to first determine whether the structure truly represents a lymph node. This initial distinction relies on a combination of clinical assessment, B-mode imaging, Doppler evaluation, and CEUS, and may require biopsy if uncertainty persists. Accurate identification of lymph node origin is fundamental, as misclassification can lead to inappropriate diagnostic pathways or delayed treatment (Figure 1).

### Imaging Correlations

**Figure 2:**
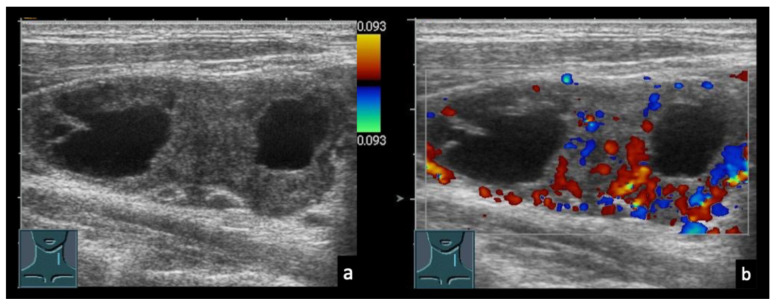
(**a**) 53-year-old patient with a history of head and neck carcinoma presenting with a left cervical lymph node containing well-defined anechoic areas. (**b**) Color Doppler sonography shows absence of detectable intranodal flow signals. Surgical lymph node excision revealed a basaloid grade 3 squamous cell carcinoma with cystic degeneration within the lymph node.

**Figure 3:**
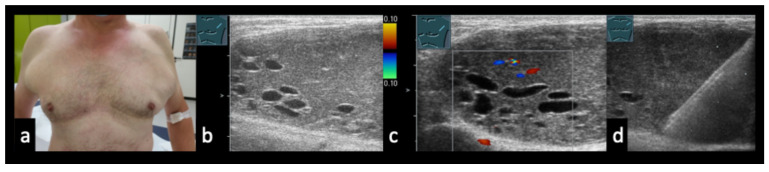
(**a**) 66-year-old patient with generalized lymphadenopathy. (**b**,**c**) B-mode ultrasound (**b**) and color Doppler sonography (**c**) show well-demarcated, cystic intranodal lesions without detectable vascularity. (**d**) Lymph node biopsy revealed lymphocytic lymphoma consistent with B-cell chronic lymphocytic leukemia (B-CLL). No specific comment was made regarding the nature of the cystic areas.

**Figure 4:**
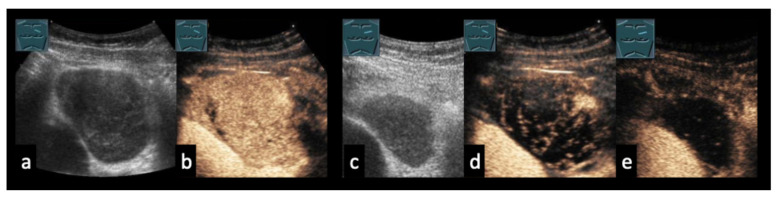
46-year-old patient with Hodgkin lymphoma before therapy (**a**,**b**) and after therapy (**c**–**e**). (**a**,**b**) Pre-therapeutic B-mode ultrasound and CEUS demonstrate a solid lymphomatous lymph node. (**c**) Post-therapy CEUS of the residual lymph node. (**d**) Early arterial phase (7 s) shows only septal enhancement. (**e**) In the parenchymal phase (13 s), no parenchymal enhancement is observed. Histology revealed a cell-poor fibrosing stromal reaction without evidence of viable tumor cells.

**Figure 5:**
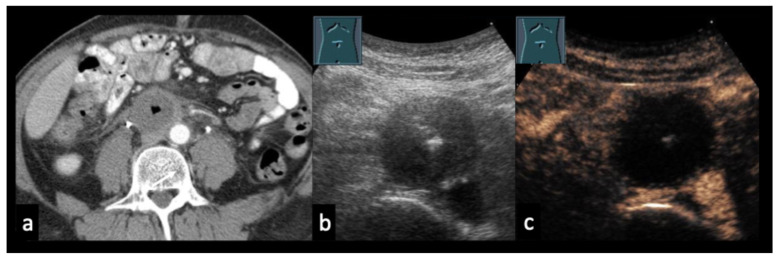
(**a**,**b**) 48-year-old patient with testicular carcinoma and paraaortic lymph node metastases after four cycles of chemotherapy. CT ((**a**), arrow) and B-mode ultrasound (**b**) demonstrate a hypoechoic residual lymph node; β-HCG remained mildly elevated at 16 U/L. (**c**): CEUS at 39 s shows absence of enhancement, indicating predominantly necrotic tissue. A residual tumor resection was planned; however, a paraaortic local recurrence developed prior to the scheduled surgery.

In the differential diagnosis, purely cystic lymph node lesions must also be considered, particularly when no imaging signs of necrosis, liquefaction, or abscess formation are present (Figure 2 and Figure 3). Furthermore, lymph node morphology can change significantly during the course of treatment. Progressive necrosis or, conversely, fibrotic transformation may develop over time, which can alter sonomorphological appearance and perfusion patterns on CEUS (Figure 4 and Figure 5).

Across these conditions, the underlying pathophysiology is manifested in characteristic sonographic appearances [6,7,17,43,44,45,46,47]:

- Liquefactive necrosis → centrally avascular cavity and peripheral rim hyperemia.
- Caseous necrosis (e.g., tuberculosis) → avascular granular core with an irregular rim, often discontinuous rim.
- Patchy immune-mediated necrosis (Kikuchi–Fujimoto, SLE) → scattered non-enhancing foci, usually without true abscess formation.
- Ischemic tumor necrosis → ill-defined non-enhancing areas within heterogeneously enhanced tumor tissue.

This close correlation between the clinical course of illness, the underlying pathophysiological processes, and their corresponding CEUS appearances may enhance diagnostic confidence, particularly in distinguishing necrotizing bacterial, granulomatous, autoimmune, and malignant lymphadenopathy. In addition, CEUS can help identify viable tissue for targeted biopsy and assist in therapeutic decision-making [9,10].

## 3. Imaging Characteristics of Necrotizing Lymphadenopathy

### 3.1. B-Mode Ultrasound

B-mode sonography is the primary imaging technique for the evaluation of lymph nodes. Necrotizing lymphadenopathy typically appears as enlarged, hypoechoic, or heterogeneous lymph nodes with loss of the fatty hilum. Liquefactive changes may manifest as central anechoic or hypoechoic regions, occasionally with fluid–fluid levels or internal debris indicating abscess formation. In tuberculosis or chronic granulomatous disease, coarse or punctate calcifications may also be present. However, these features are not pathognomonic and may overlap with cystic metastatic nodes or necrotic malignant nodes, thereby limiting the specificity of B-mode ultrasound.

### 3.2. Color and Power Doppler Imaging

Doppler ultrasound provides complementary information on nodal vascularity. In necrotizing lymphadenopathy, central vascular flow is typically reduced or absent, while peripheral hypervascularity may occur due to inflammatory hyperemia, producing the characteristic “rim sign”. However, Doppler techniques have limited sensitivity for slow or microvascular flow, and vascular signals may be attenuated in deeply located nodes or obscured by technical artifacts. Consequently, Doppler alone is insufficient for reliably distinguishing necrotic infection from malignant necrosis, as only CEUS can confirm the absence of perfusion with higher sensitivity [48].

### 3.3. Contrast-Enhanced Ultrasound (CEUS)

CEUS considerably enhances the diagnostic assessment of necrotizing lymphadenopathy by providing real-time visualization of nodal microvascular perfusion. Its main advantages include high sensitivity for detecting microvascular flow, the ability to delineate viable from non-viable tissue, and precise delineation of abscess cavities [8,17,49,50].

Typical CEUS patterns include:

- Abscesses: central non-enhancement corresponding to liquefaction, surrounded by a regular peripheral rim of hyperenhancement.
- Tuberculous or atypical mycobacterial necrosis: avascular central core with irregular or discontinuous peripheral enhancement reflecting granulomatous tissue.
- Autoimmune necrotizing lymphadenopathy (e.g., Kikuchi–Fujimoto, SLE): patchy or scattered non-enhancing foci without true liquefied cavities.
- Malignant necrosis (lymphoma, metastatic carcinoma): heterogeneous enhancement with ill-defined, irregular non-enhancing areas within heterogeneously enhanced tumor parenchyma.

Recognition of these enhancement patterns may improve diagnostic confidence, support selection of viable areas for biopsy, and help avoid unnecessary or inappropriate interventions. The illustrated CEUS patterns are not disease-specific and may overlap with infectious, granulomatous, or malignant lymphadenopathy.

### 3.4. Cross-Sectional Imaging Correlation

CT and MRI can demonstrate nodal necrosis with high anatomical detail but lack the real-time perfusion detail of CEUS. In many cases, CEUS offers greater bedside practicality, avoids ionizing radiation or nephrotoxic contrast agents, and can be performed repeatedly without risk, which is particularly advantageous for pediatric patients, pregnant women, or individuals requiring serial follow-up examinations.

### 3.5. Case Vignettes (Figure 6, Figure 7, Figure 8, Figure 9, Figure 10, Figure 11, Figure 12, Figure 13, Figure 14, Figure 15, Figure 16, Figure 17 and Figure 18)

The following clinical vignettes illustrate representative CEUS patterns in necrotizing lymphadenopathy. To enhance readability, descriptions focus primarily on the key distinguishing imaging and clinical features of each case.

**Figure 6:**
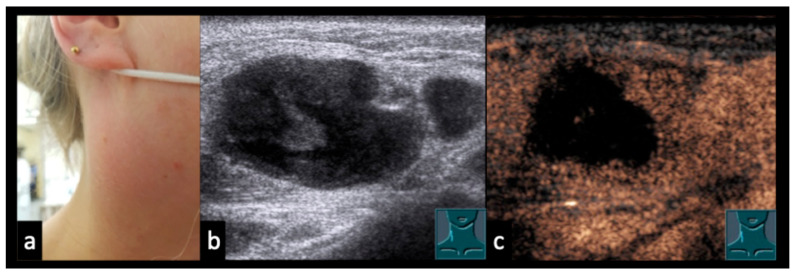
32-year-old patient with a swollen and reddened throat (**a**), undergoing immunosuppressive therapy for Crohn’s disease and presenting with abscessing lymphadenopathy without detectable bacterial pathogens. (**b**): B-mode ultrasound shows an inhomogeneous lymph node. (**c**): CEUS demonstrates partial absence of enhancement, consistent with abscess formation.

**Figure 7:**
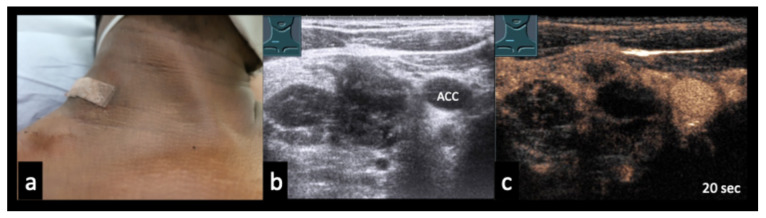
30-year-old patient with confirmed cervical lymph node tuberculosis (**a**). (**b**) B-mode ultrasound shows an inhomogeneous conglomerate lymph node mass. (**c**): CEUS demonstrates partial absence of enhancement, consistent with necrotizing tuberculous lymphadenopathy. ACC = common carotid artery.

**Figure 8:**
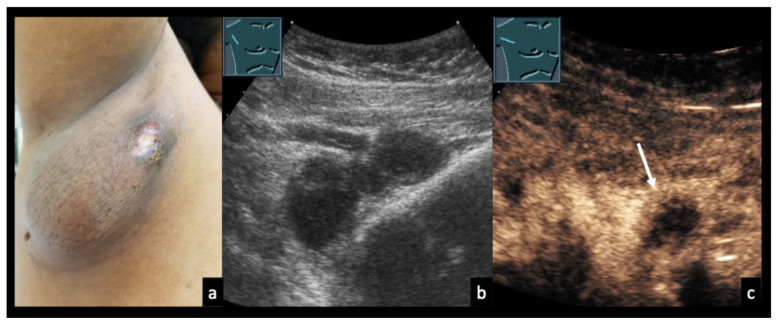
31-year-old female patient with confirmed right axillary lymph node tuberculosis (**a**). (**b**) B-mode ultrasound shows heterogeneous lymph nodes. (**c**) CEUS demonstrates partially absent enhancement (arrow), consistent with necrotizing tuberculous lymphadenopathy.

**Figure 9:**
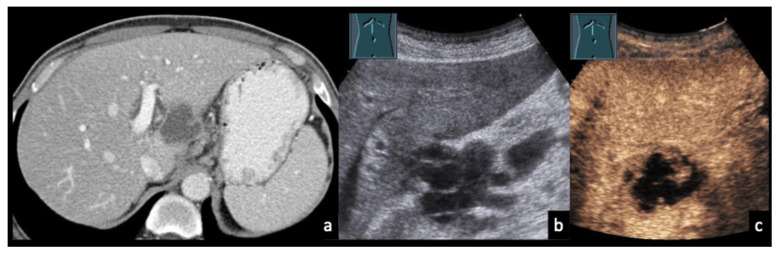
57-year-old male patient with confirmed lymph node tuberculosis in the hepatic hilum ((**a**), CT). (**b**) B-mode ultrasound demonstrates multiple partially anechoic lymph nodes. (**c**) CEUS shows a large non-enhancing area, consistent with extensive necrotic transformation.

**Figure 10:**
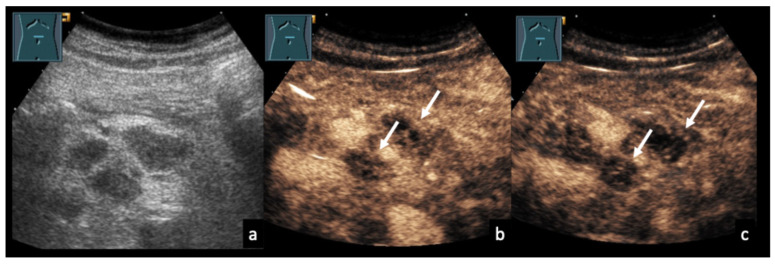
Mesenteric lymphadenopathy due to tuberculosis. 68-year-old male patient with confirmed mesenteric lymph node tuberculosis (**a**). (**b**,**c**) On CEUS at 54 and 90 s, the mesenteric lymph nodes show heterogeneous enhancement with focal areas of absent contrast uptake (arrows).

**Figure 11:**
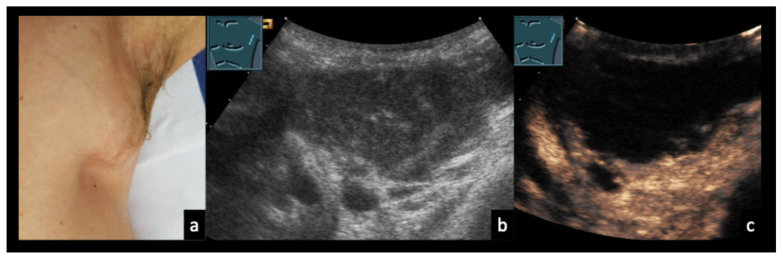
34-year-old male patient (**a**) with confirmed axillary lymph node tularemia (*Francisella tularensis*). (**b**) B-mode ultrasound shows heterogeneous, confluent lymph node formations. (**c**) On CEUS at 40 s, there is near-complete absence of enhancement, consistent with abscess formation.

**Figure 12:**
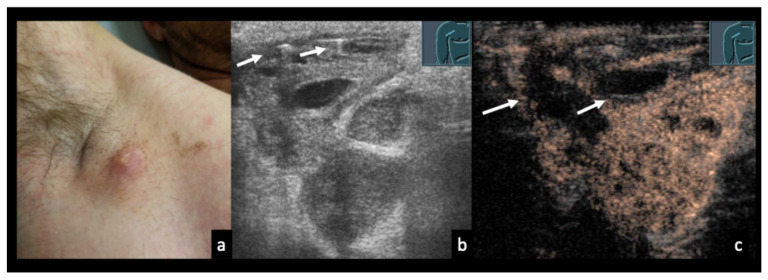
31-year-old female patient (**a**) with confirmed localized axillary lymph node infection due to *Bartonella henselae* (cat-scratch disease). (**b**) B-mode ultrasound shows heterogeneous, confluent lymph nodes with intranodal gas reflections (arrows). (**c**) On contrast-enhanced ultrasound (CEUS), the lymph nodes demonstrate partially absent enhancement (arrows), consistent with abscessing lymphadenopathy.

**Figure 13:**
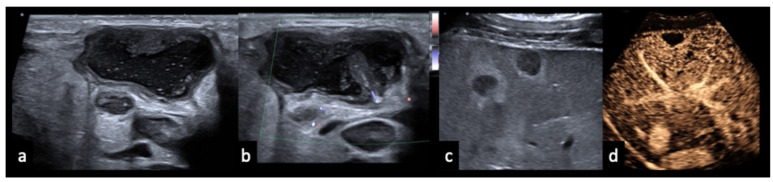
23-year-old male patient presenting with weakness, shortness of breath, and painful cervical lymph node swelling. (**a**) B-mode ultrasound shows a nearly anechoic cervical mass with floating internal echoes. (**b**) Color Doppler imaging demonstrates absence of intralesional vascularity. (**c**) Abdominal B-mode ultrasound reveals multiple hepatic lesions with varying morphology. (**d**) CEUS shows perilesional hyperperfusion in the arterial phase (30 s), while most of the mass appears non-enhancing, consistent with extensive necrosis. The final diagnosis of disseminated bartonellosis was established by serology. During medical therapy with doxycycline and rifampicin, the cervical abscess spontaneously perforated through the skin. Shortness of breath was attributed to acute myocarditis.

**Figure 14:**
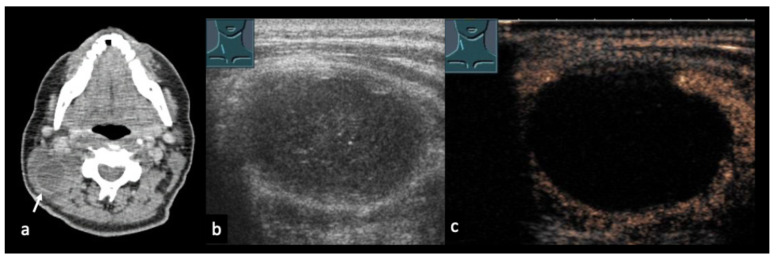
50-year-old female patient with follicular lymphoma at initial diagnosis, demonstrated on CT ((**a**), arrow). (**b**) B-mode ultrasound shows a homogeneously hypoechoic lymph node. (**c**) On CEUS in the early phase (8 s), the lymph node shows only peripheral rim enhancement. Histology revealed an infiltrate of a low-grade B-cell non-Hodgkin lymphoma, consistent with the clinically known follicular lymphoma, with partial necrosis and associated chronic granulating and fibrosing inflammation.

**Figure 15:**
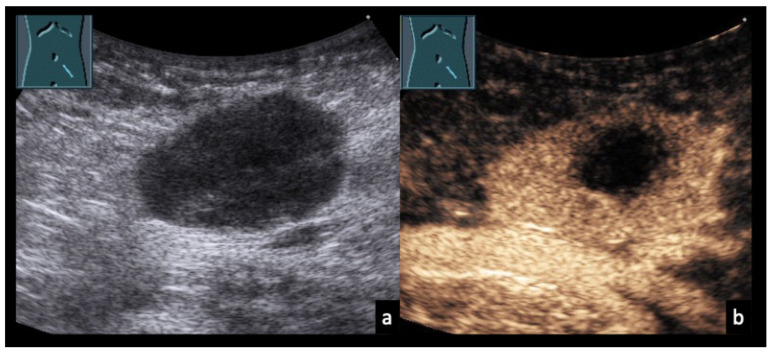
49-year-old male patient with grade 1 follicular lymphoma prior to therapy. (**a**) B-mode ultrasound shows a predominantly hypoechoic inguinal lymph node. (**b**) On CEUS, there is central absence of enhancement, most consistent with necrotic transformation.

**Figure 16:**
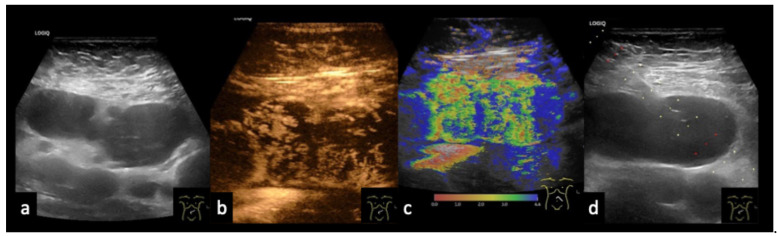
Diffuse large B-cell non-Hodgkin lymphoma. 70-year-old male patient presenting with weight loss and non-specific abdominal complaints. (**a**) B-mode ultrasound shows multiple round hypoechoic abdominal masses measuring up to 35 mm. (**b**) On CEUS, peripheral inhomogeneous enhancement is observed, while the central portions show no enhancement. (**c**) The temporal enhancement pattern is demonstrated by parametric imaging. (**d**) Histologic confirmation was obtained by percutaneous, ultrasound-guided biopsy.

**Figure 17:**
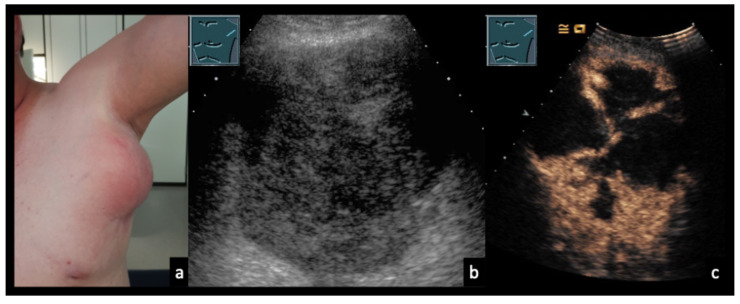
67-year-old male patient (**a**) with confirmed left axillary lymph node metastasis from malignant melanoma. (**b**) B-mode ultrasound shows an enlarged, heterogeneous lymph node. (**c**) On CEUS, there is partial absence of enhancement, consistent with necrotic transformation.

**Figure 18:**
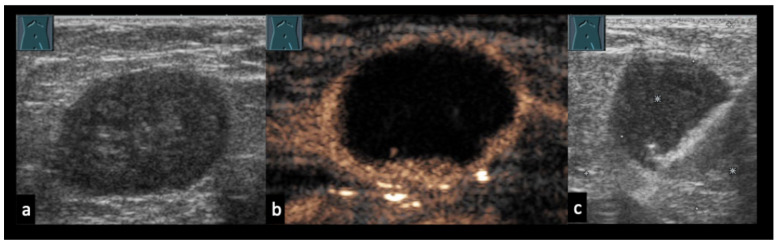
64-year-old female patient with vulvar carcinoma and a hypoechoic lymph node on B-mode ultrasound (**a**). (**b**) On CEUS at 20 s, the lymph node shows only peripheral rim enhancement. (**c**) Fine-needle aspiration revealed a basaloid squamous cell carcinoma with HPV association.

## 4. Review of the Literature

The currently available literature on CEUS in necrotizing lymphadenopathy is heterogeneous and largely observational. Most publications consist of single-center retrospective studies, small prospective cohorts, or case reports and case series. Sample sizes are frequently limited, and only a small number of studies include comparative analyses with other imaging modalities or histopathologic reference standards. Consequently, the strength of evidence remains moderate to low. The most substantial data currently exist for tuberculous lymphadenopathy and malignant lymph nodes, whereas evidence for zoonotic infections, autoimmune necrotizing lymphadenopathy, and rare infectious causes is mainly derived from individual case reports or small case series. Therefore, many of the described CEUS patterns should be interpreted as descriptive observations rather than validated diagnostic criteria.

### 4.1. CEUS for Necrotizing/Abscessing Lymphadenopathy—Overall Evidence

Contrast-enhanced ultrasound (CEUS) has proven highly valuable in evaluating necrotizing lymphadenopathy. Several groups have reported that CEUS may improve sensitivity for detecting nodal necrosis compared with B-mode and Doppler [6,7,49,51,52,53,54]. Similar CEUS patterns may be encountered in both benign and malignant necrotizing lymphadenopathy; definitive diagnosis requires clinicopathologic correlation.

### 4.2. Tuberculous and Atypical Mycobacterial Lymphadenopathy

In cervical tuberculous lymphadenopathy, Möller et al. and Zhao et al. described absent central enhancement with irregular rim vascularity as the CEUS hallmark [15,55]. Li et al. showed that CEUS guidance during core-needle biopsy significantly increased diagnostic yield by avoiding necrotic regions [14]. Yu et al. demonstrated the role of multimodal ultrasound including CEUS, for monitoring therapy response, correlating perfusion recovery with clinical improvement [56]. Irregular rim enhancement surrounding an avascular core is frequently seen in tuberculous lymphadenopathy but may overlap with necrotic squamous cell carcinoma metastases or post-therapeutic malignant nodes.

### 4.3. Bacterial Lymphadenopathy

Pyogenic bacterial nodes typically show complete non-enhancement centrally and smooth rim enhancement. Cui et al. [57] highlighted the diagnostic accuracy of CEUS in identifying abscesses, particularly when Doppler failed to show central flow. Although smooth peripheral rim enhancement with central non-perfusion is typical of abscesses, similar patterns may also be observed in necrotic metastatic lymph nodes or treated lymphoma.

### 4.4. Cat-Scratch Disease (Bartonella henselae)

Ridder-Schröter et al. reported abscess-forming lymphadenopathy with nearly identical CEUS features to those of pyogenic infection [58]. More recently, Bruni et al. described atypical sonographic manifestations in a child with cat-scratch disease, stressing the usefulness of perfusion imaging in atypical cases [59].

### 4.5. Tularemia (Francisella tularensis)

Tularemia often leads to abscessing cervical lymphadenopathy. Maurin and Gyuranecz (2016, Lancet Infectious Diseases) reviewed the European experience, describing abscess formation as a common clinical presentation [24], while Sjöstedt outlined the underlying pathophysiology that explains the CEUS appearance of an avascular necrotic core and rim hyperemia [60].

### 4.6. Yersinia enterocolitica/Pseudotuberculosis

Mesenteric adenitis due to Yersinia can mimic appendicitis. Bottone described the pathological correlates [61], while Nuorti et al. documented outbreaks with abscessing lymphadenopathy, which on CEUS manifests as focal non-enhancing areas among otherwise reactive nodes [62].

### 4.7. Brucellosis

Franco et al. reviewed the clinical spectrum, noting that although granulomatous lymphadenopathy predominates, necrosis may occur [63]. In such cases, CEUS typically demonstrates patchy avascular regions within otherwise perfused nodal tissue.

The described (non-)enhancement patterns observed in zoonotic lymphadenopathy are not specific and may be indistinguishable from pyogenic abscesses or malignant necrosis without clinical and serological correlation.

### 4.8. Opportunistic Fungal Infections

Wheat et al. detailed nodal involvement in histoplasmosis [64], while Perfect et al. outlined cryptococcal disease [65]. Both can cause necrotizing nodes in immunocompromised patients; CEUS depicts avascular foci with irregular borders, sometimes indistinguishable from mycobacterial disease without biopsy.

### 4.9. Toxoplasmosis and Leishmaniasis

Montoya and Liesenfeld described toxoplasmosis, where necrosis is rare but possible in severe cases [66]. Alvar et al. highlighted that advanced leishmaniasis may involve necrotizing lymphadenopathy [67]. CEUS usually shows homogeneous enhancement, with necrotizing foci being uncommon and non-specific.

### 4.10. Autoimmune Necrotizing Lymphadenopathy

Perry and Choi reviewed Kikuchi–Fujimoto disease, emphasizing apoptotic necrosis without neutrophils [40]. On CEUS this results in patchy perfusion defects rather than fluid-filled abscess. Similarly, lupus lymphadenopathy may show heterogeneous perfusion defects due to immune complex vasculitis. Patchy or scattered non-enhancing areas may mimic early malignant necrosis or partially necrotic infection, and CEUS alone cannot reliably establish the diagnosis.

### 4.11. Malignant Lymph Nodes with Necrosis

In oncology, CEUS can help distinguish tumor necrosis from infection. Liu et al. demonstrated that post-vascular phase CEUS improves detection of viable tissue in small metastatic cervical nodes [68]. Saito and Shiga summarized sonographic features of metastatic nodes, including irregular central necrosis [69]. Compared with abscesses, malignant necrosis typically shows ill-defined non-enhancing areas embedded within enhancing tumor tissue. Heterogeneous enhancement with ill-defined non-perfused areas is suggestive but not specific for malignancy, as similar findings may occur in advanced infection or granulomatous disease.

### 4.12. Clinical Implications

CEUS may support differentiation between abscessing nodes, malignant necrosis, and inflammatory conditions. It may increase diagnostic confidence by providing real-time visualization of microvascular perfusion [Table 1].

### 4.13. Therapeutic and Follow-Up

CEUS also facilitates ultrasound-guided aspiration or drainage by helping to identify viable and accessible target areas. It also assists in avoiding necrotic regions during core-needle biopsy, thereby improving diagnostic yield.

Follow-up: CEUS can also be used for follow-up to monitor therapy response, such as resolution of abscess cavities. Its lack of ionizing radiation and nephrotoxic contrast agents makes it suitable for repeated examinations.

A diagnostic algorithm for necrotizing lymphadenopathy is shown below.

**Diagnostic** **algorithm for necrotizing lymphadenopathy**

1. Identify lesion origin

Confirm lymph node origin (exclude cysts, hernia, seroma, pseudoaneurysm).

2. Baseline ultrasound

B-mode + Doppler assessment of size, morphology, vascularity

3. CEUS assessment

Is there central non-enhancement?

  **Yes** → necrosis or abscess likely

  **No** → consider solid/inflammatory lymphadenopathy

4. Pattern recognition on CEUS

Smooth rim + complete avascular center → abscess

Irregular/discontinuous rim → granulomatous disease (e.g., TB)

Patchy perfusion defects → autoimmune necrotizing lymphadenopathy

Heterogeneous enhancement with ill-defined necrosis → malignancy

5. Clinical integration

Infection signs, immune status, malignancy history, therapy status

6. Targeted intervention

CEUS-guided biopsy (avoid necrosis)

Aspiration/drainage if abscess suspected

Cross-sectional imaging if deep or equivocal

## 5. Diagnostic Pitfalls in CEUS Assessment of Necrotizing Lymphadenopathy

Despite its ability to depict microvascular perfusion in real time, CEUS is subject to several diagnostic pitfalls that must be recognized to avoid misinterpretation. The most important limitation is the substantial overlap of qualitative enhancement patterns among infectious, granulomatous, autoimmune, and malignant lymphadenopathy. Features such as peripheral rim enhancement, central non-perfusion, internal septations, and heterogeneous enhancement are not disease-specific and may occur across different etiologies.

Necrotic malignancy versus abscessing infection represents a frequent diagnostic challenge. Smooth rim enhancement with central non-enhancement, typically associated with abscess formation, may also be observed in necrotic metastatic lymph nodes or in lymphomas after chemotherapy or radiotherapy. Conversely, irregular or discontinuous rim enhancement, often linked to granulomatous disease such as tuberculosis, can closely resemble necrotic squamous cell carcinoma metastases.

Autoimmune necrotizing lymphadenopathy, particularly Kikuchi–Fujimoto disease or lupus lymphadenopathy, may show patchy or scattered perfusion defects on CEUS that can mimic early malignant necrosis or partially treated infection. The absence of true liquefaction or pus cannot always be reliably determined by CEUS alone.

Post-therapeutic changes constitute another important pitfall. Fibrosis, treatment-induced necrosis, and inflammatory remodeling may result in absent or markedly reduced enhancement, potentially leading to false assumptions of residual active disease or, conversely, complete remission.

Technical and operator-related factors may further confound interpretation. Excessive transducer pressure, inappropriate gain or focal zone settings, motion artifacts, or premature microbubble destruction can create apparent perfusion defects that mimic necrosis.

Given these pitfalls, CEUS findings should always be interpreted in conjunction with clinical presentation, laboratory results, treatment history, and, when necessary, histopathological confirmation. CEUS should be regarded as a tool that enhances diagnostic confidence and procedural guidance rather than a stand-alone method for definitive etiologic classification.

## 6. Limitations

Although CEUS has demonstrated significant advantages in the assessment of necrotizing and abscessing lymphadenopathy, several limitations need to be considered.

### 6.1. Operator Dependence and Technical Variability

CEUS is highly operator dependent, and the quality of image acquisition and interpretation varies with examiner experience. Differences in contrast administration technique, mechanical index settings, and timing of vascular-phase imaging can affect reproducibility.

### 6.2. Restricted Penetration

Ultrasound penetration is limited in obese patients, in the cases of deep-seated lymphadenopathy (e.g., mediastinal or retroperitoneal), or when overlying bowel gas interferes with acoustic windows. In such cases, cross-sectional imaging with CT or MRI may still be required.

### 6.3. Limited Standardization

Unlike liver CEUS (where LI-RADS provides structured criteria), no universally accepted classification system exists for lymph node CEUS. The interpretation of enhancement patterns, regular versus irregular rim enhancement, percentage of necrosis, or heterogeneity—remains somewhat subjective, limiting multicenter comparability.

### 6.4. Overlap of Imaging Features

Certain benign and malignant conditions can present with overlapping CEUS findings. For example, irregular necrotic patterns can be seen in both tuberculous lymph nodes and metastatic carcinoma. In these cases, CEUS alone cannot establish a definitive diagnosis and should be complemented by clinical data and histopathology.

### 6.5. Limited Disease-Specific Evidence

Most published studies focus on tuberculous lymphadenopathy or malignant lymph nodes. Data on less common causes, such as tularemia, brucellosis, yersiniosis, leishmaniasis, and autoimmune disorders, are often limited to case reports or small series, reducing generalizability.

### 6.6. Requirement for Intravenous Contrast Administration

Although ultrasound contrast agents (UCAs) have an excellent safety profile, they remain contraindicated in rare settings (e.g., severe cardiopulmonary shunts, allergy to their components). Availability may also be limited in certain regions due to regulatory or cost issues.

#### Research Gaps

Quantitative CEUS analysis (e.g., perfusion kinetics, time–intensity curves) is still underused. Multicenter prospective studies comparing CEUS with CT, MRI, or PET/CT are still lacking. Furthermore, there is little evidence on standardized CEUS-guided therapeutic algorithms for lymphadenopathy.

### 6.7. Future Directions

The growing evidence base for CEUS in necrotizing lymphadenopathy underscores its potential to become a standard component of nodal imaging. To achieve this, several areas deserve attention.

**Development of standardized CEUS criteria:** While CEUS features such as rim enhancement, central non-perfusion, and heterogeneous enhancement are well recognized, there is no consensus classification for lymph nodes comparable to the CEUS LI-RADS system in hepatology. The creation of reproducible scoring systems could harmonize interpretation across centers and facilitate multicenter trials.

**Quantitative and parametric analysis:** Most studies rely on qualitative interpretation of enhancement patterns. Perfusion quantification using time–intensity curves, wash-in/wash-out kinetics, and parametric perfusion maps could reduce subjectivity, improve diagnostic accuracy, and support AI-based analysis tools.

**Integration with multiparametric ultrasound:** Combining CEUS with elastography, high-frequency B-mode, and Doppler may provide a comprehensive “multiparametric” assessment of lymph nodes. Such integrated protocols could further differentiate infection, autoimmune necrosis, and malignant infiltration.

**CEUS-guided interventional strategies:** Although CEUS guidance for biopsy and drainage has been shown to increase diagnostic yield, standardized algorithms for procedural guidance are still lacking. Future research should focus on outcome-based comparisons of CEUS-guided versus conventional biopsy or aspiration.

**Pediatric and vulnerable populations:** Because CEUS avoids radiation and nephrotoxic contrast agents, it is especially suited for children, pregnant women, and patients requiring repeated follow-up. Dedicated pediatric trials could establish CEUS as first-line imaging modality in this demographic.

**Artificial intelligence and machine learning:** Automated pattern recognition and quantitative CEUS analysis through AI could enhance reproducibility and support decision-making in equivocal cases. Integration of CEUS imaging into multimodal AI frameworks may help predict underlying etiology and optimize treatment planning.

**Comparative data between CEUS and CT, MRI, or PET-CT** in necrotizing lymphadenopathy remain limited and largely indirect. Available evidence suggests that CEUS complements cross-sectional imaging by providing real-time microvascular perfusion assessment and improved delineation of viable versus necrotic tissue, particularly for biopsy guidance. Quantitative CEUS approaches, including time–intensity curve analysis, have shown promising results in small series, mainly in tuberculous and metastatic lymph nodes, but require further standardization and multicenter validation [57,70,71,72].

**Multicenter prospective validation:** Large-scale studies directly comparing CEUS to CT, MRI, and PET/CT across diverse disease etiologies are needed to define diagnostic accuracy, cost-effectiveness, and patient outcomes. Such evidence will be crucial for wider clinical adoption and inclusion in diagnostic guidelines.

## 7. Conclusions

Necrotizing (abscessing) lymphadenopathy represents a diagnostic challenge due to its diverse etiologies, ranging from bacterial and mycobacterial infections to autoimmune disorders and malignant tumors. While B-mode ultrasound and Doppler provide important initial information, their specificity in differentiating necrotic from viable tissue is limited. CEUS may add relevant diagnostic information by enabling real-time assessment of microvascular perfusion, allowing more precise delineation of abscess cavities, caseous necrosis, patchy immune-mediated defects, and malignant necrosis.

The available literature consistently demonstrates that CEUS may improve diagnostic confidence, guides biopsy and drainage procedures, and facilitates safe follow-up, particularly in pediatric and vulnerable populations. However, current evidence remains heterogeneous and largely focused on tuberculous lymphadenopathy and malignant lymph nodes, with limited data for rarer infectious and autoimmune conditions.

Future research should prioritize standardized CEUS criteria, quantitative and AI-supported analysis, integration into multiparametric ultrasound protocols, and multicenter validation studies. With these advances, CEUS has the potential to become a cornerstone technique for the non-invasive evaluation and management of necrotizing lymphadenopathy, bridging the gap between structural imaging and functional, pathology-oriented assessment.

Clinical Take-Home Messages: In clinical practice, CEUS should be regarded as a complementary tool within a multiparametric ultrasound approach rather than a stand-alone diagnostic method. Recognition of enhancement patterns, such as central non-enhancement with rim hyperemia, irregular avascular cores, or heterogeneous perfusion defects, may help identify necrosis, guide targeted biopsy, and support clinical decision-making. However, substantial overlap exists between infectious, autoimmune, and malignant etiologies, and definitive diagnosis frequently requires clinicopathological correlation.

## Figures and Tables

**Table 1:** **Multiparametric** **ultrasound features of necrotizing lymphadenopathy: typical CEUS patterns, diagnostic overlap, and recommended next steps.**

Etiology	B-Mode US	Doppler	CEUS Pattern	Key Diagnostic Overlap/Pitfall	Next Step
Pyogenic infection	Enlarged, hypoechoic; liquefaction	Peripheral flow	Central non-enhancement, smooth rim	Necrotic metastasis	Aspiration/drainage
Tuberculosis/atypical mycobacteria	Heterogeneous; calcifications	Reduced central flow	Avascular core, irregular rim	Metastatic SCC	CEUS-guided biopsy
Zoonotic infection	Confluent, heterogeneous nodes	Variable	Marked central non-enhancement	Pyogenic abscess	Serology ± aspiration
Autoimmune (Kikuchi, SLE)	Hypoechoic, no true cavity	Reduced flow	Patchy non-enhancing areas	Early malignancy	Histologic confirmation
Malignancy	Round, hilum loss	Chaotic/peripheral	Heterogeneous enhancement with necrosis	Infection	Targeted biopsy

## Data Availability

No new data were created or analyzed in this study.
